# The Integration of People Convicted of a Sexual Offence Into the Community and Their (Risk) Management

**DOI:** 10.1007/s11920-021-01258-4

**Published:** 2021-07-01

**Authors:** K. F. McCartan, K. Richards

**Affiliations:** 1 University of the West OF England, Bristol, UK; 2 Queensland University of Technology, Brisbane, Australia

**Keywords:** People who have sexually offended, Community integration, Risk management, Multi-disciplinary approaches, Multi-agency approaches, Epidemiological criminology

## Abstract

**Purpose of Review:**

We are reviewing recent research into the community integration of men convicted of a sexual offence and their (risk) management. This is a high-profile political issue that binds together research in psychology, criminology, politics, health, public health, and policy studies. The review will demonstrate that a multi-disciplinary, life course, EpiCrim-oriented approach is the most effective way of reducing re-offending and promoting desistance in this population.

**Recent Findings:**

Research demonstrates that life course development, especially from psychology and criminology, has an impact on whether people sexually offend or not. Therefore, to understand sexual offending behaviour, we need to look at the aetiology of said behaviour from a nature and a nurture perspective. Therefore, we need to use an Epidemiological Criminology (a marriage of Public Health and criminology) approach that works at all four stages of the Socio-Ecological Model (SEM) (individual, interrelationship, community, and societal). The research encourages a person first approach, that we look at Adverse Childhood Experiences and past trauma in the lives of men who sexually offend and use this, in conjunction with strength-based approaches, to inclusively integrate them into society.

**Summary:**

The prevention of sexual offending, both first time offending, and relapse prevention require a multi-level, multi-disciplinary approach. Successful desistance from sexual offending is as much about the community and society as it is about the individual.

## Introduction

Sexual abuse and sexual offending are high level socio-political, policy, media, and community issues [1–3] which transverse all levels of society and are present across all countries [4–9]. Since the 1970s [10, 11], arguably the major starting point for research into sexual abuse, we have seen the emergence of several narratives linked to sexual abuse and how best to respond to it [1, 12••]. In today’s socio-political climate, even before the advent of the *#metoo* movement [13], sexual offending was viewed as a high-profile issue that factored in political debates [14–16], media coverage [17–20], and public discourses [3, 18, 21–24]. Often these public debates were, and still are, punitive in nature [25–28, 29•] and viewed within a risk management framework [11, 15, 30]. In recent years, this punitive discourse around sexual offences has started to shift with the introduction of public health [21, 31–34] and life-course [35–39] perspectives that reinforce strengths-based approaches [40, 41•, 42] and desistance pathways [28••, 43, 44]. This article discusses how the incorporation of public health approaches is starting to change the policy, practice, and messaging around the community integration of people who have sexually offended.

## Definitional Challenges and Their Impact on Sexual Abuse

It is important to understand that defining sexual offences is complex and can vary widely within and among different countries as well as legal jurisdictions [3, 45–48]. This legal complexity is further compounded by the context within which the offence happens, the nature of the offence, the impact upon the victim/survivor, and the demographics of the person committing it [1, 12••]. This means that sentencing and related outcomes [i.e. incarceration, treatment] are case-dependent and varied. This is problematic as it can result in the perception that the criminal justice response to sexual offending is inconsistent and not fit for purpose [49–53]. Unfortunately, this works to erode trust in the criminal justice system by victim/survivors [49, 54–57], which has resulted in an increase in the under-reporting of sexual offences [54]. Determining the true scale of sexual offending is therefore difficult. While it is difficult to determine the actual prevalence of sexual abuse, research indicates that it is higher compared to official data. [58]. Prevalence studies indicate that, in Public Health terms, sexual abuse is an epidemic [21], and that the people who commit sexual offences are less likely to be abnormal “monsters” and more likely to be likely to be representative members of society [28••].

## Current Approaches to Community Risk Management

Current responses to sexual offending are rooted in the criminal justice processes [15, 46, 59]. Traditionally, most individuals convicted of a serious sexual offence will receive a prison sentence, or a suspended prison sentence to be served in the community, in conjunction with a treatment or rehabilitation programme tied to the nature of their offence and the risk they pose [9, 60, 61]. Rehabilitation is based on the underlying principle that people can and want to change and is delivered though a Cognitive-Behavioural framework that uses strength-based models and pro-social behaviour modelling [1, 12••, 62, 63••, 64–68]. In recent years, we have seen a de-pathologising of people who commit sexual offences as it is no longer accepted that all people who commit sexual offences have a severe mental illness [69–71]. Instead, we are seeing that inclusion IN treatment programmes is being determined not by their offence but instead by level of anti-sociality, any co-morbidities and risk of re-offending [69–71]. This emphasises that rehabilitation needs to be bespoke to the individual [72–76]. However, paradoxically, we see punitive criminal justice policies, often replicated internationally [3, 15, 46, 77], that run contrary to the desistance and harm reduction messages of rehabilitation [28••]. These harmful and problematic policies include the use of polygraph testing [78, 79], registration and/or community notification policies [80–82], housing restrictions [83, 84], and limitations on employment [85, 86]. The reason why these policies can reduce the successful community integration of people convicted of a sexual offence is that they identify them as an “other”, limit integration, and reduce said individuals’ opportunity to change. The reality is that in attempting to protect the community and safeguard citizens, these policies often increase risk rather than reduce it [28••, 44, 68, 87]. This is clearly evidenced by the fact that prolonged community inclusion reduces risk longitudinally [1]. We are starting to see a growth in community-based and community-driven interventions for people convicted of sexual offences that are rooted in the desistance framework, including the increased use of restorative justice [88, 89], and Circles of Support and Accountability [90, 91]. Although, a lot of new community interventions are developed and implemented based on professional knowledge and expertise, they need more research and evaluation before they can be determined to be effective in supporting community integration. In addition to helping with the rehabilitation and management of people convicted of a sexual offence, these innovations in community integration interventions help improve the public’s knowledge on sexual abuse and therefore improve public attitudes to and openness for community integration [92].

## Understanding the Role and Function of Strengths-Based Approaches in Integrating People Convicted of a Sexual Offence

In recent years, we have seen continued growth in strengths-based approach to the treatment and rehabilitation of people convicted of a sexual offence, with increased focus on the Good Lives Model and pro-social inclusion [1, 12••, 63••, 64, 65, 72–74]. The strengths-based approach to rehabilitation reinforces that rehabilitation is a process rather than an outcome [28••]. This reinforces that behaviour change is learnt and that Cognitive-Behavioural approaches, in which most programmes/interventions for individuals convicted of a sexual offence are rooted, are a logical way forward [93, 94]. A strengths-based approach is important in rehabilitation because it emphasises that a person is more than their offence, that they have strengths as well as deficits, and that by building upon these strengths they can change their problematic behaviour; therefore, increasing their ability to successfully integrate into the community.

## Using a Multi-disciplinary Approach to Understand Sexual Offending

In responding to sexual abuse, we need to examine the behaviour of the person who committed it from several perspectives [95], which means considering their psychology, wellbeing, mental health, family and peer relationships, employment, education, lifestyle, and socio-demographic factors [1, 46, 96, 97]. Evidence indicates that of people who commit sexual offences are not radically different from individuals who commit other offences [98], and that we need better understand the impact of developmental and social factors, important factors that highlight the impact of nature and nurture, on the lives people who commit sexual offences [3, 15]. Therefore, we need to better unify psychological, social, health, and wellbeing research in this arena [99]. One method for doing this is by using an Epidemiological Criminology (EpiCrim) approach [100, 101]. EpiCrim understands and responds to crime at a population level by using public health approaches that work at an individual, interpersonal, community, and societal level across all four stages of prevention (primary, secondary, tertiary, and quaternary) [10, 98, 102]. An EpiCrim approach emphasises the importance of individual, their relationships, and wider social context so that we can better understand their pathways into offending as well as develop fit for purpose rehabilitation programmes. The EpiCrim approach is starting to be used in the field of sexual offender risk management and community integration, [10, 42, 98, 102–104]]. Additionally, taking an EpiCrim approach has moved the narrative around community integration and risk management away from a purely professional one to a more community based one with increased education [10, 102], bystander intervention training [105–107], and community integration programmes/interventions [108–110]. An EpiCrim approach reinforces that sexual abuse is a community as well as an in individual issue, and therefore needs an integrated, multi-faceted community response.

## From Multi-Disciplinary to Multi-Agency Approaches: Theory to Practice

One cannot take a siloed approach to understanding the aetiology, process, treatment, and/or management of people convicted of a sexual offence. Instead, we need a multi-disciplinary approach that emphasises that sexual offending, like all offending, is created from a series of different “events” or “processes” in a person’s life (i.e. what works approach—111). Effective community integration and risk management needs an individualised approach that includes all the relevant agencies and organisations who work with the service user [41•, 45, 112–115]. Hence, we need to put the person who committed the sexual offence at the heart of the rehabilitation and community integration process [68, 116–118]. Not only does a bespoke EpiCrim approach to community integration help in the prevention of reoffending, but it can also help in the prevention of first time offending by giving us insight into the way that individuals think about their offending, their pathways into it, and it allows us to hear from the service user—potentially the most effective means of intervening.

## What “Successful” Community Integration Looks Like

One of the main challenges in the community integration of people convicted of a sexual offence is articulating what success looks like. Community management policies are impacted by Key Performance Indicators (KPIs) related to the criminal justice organisations, charities, and non-government organizations that must execute them. Generally, success is measured in terms of “re-conviction” rather than “re-offending” [119, 120] or “harm reduction” [74, 102, 121]. This is problematic because someone can re-offend and not get caught. Further, individuals may re-offend at a lower rate, or in a different way, from their original offending behaviour [62, 63••]. Therefore, the question must be asked: is there a better way of identifying risk, managing risk, and measuring success? Should we be looking at harm reduction and desistance [28••, 43, 44, 87] as a means of identifying continued, or changing, risk [30, 119, 122, 123]?

If we are serious about sexual offending being a multi-disciplinary and multi-agency issue, we need to better understand how other organisations, and fields, develop and evidence their KPI’s so that we can make ours fit for purpose. This means that we should look at success criteria in violence prevention [124], addiction studies [125], and health [126], among other fields, and build a new, more tailored approach to measuring success. People who commit sexual offences generally have low re-conviction rates [62, 63••, 127], are compliant with community management strategies [28••, 128, 129], and those who do re-offend commonly do so at a lower level than their original offences [62, 63••]. Hence, we are starting from a low base rate and trying to understand why a smaller population goes on to reoffend while the larger group does not. Therefore, methodologically, qualitative research and case studies work better in desistance focused research projects as we want to better understand individual behaviours to build more effective interventions. However, these approaches offer methodological challenges in many innovative interventions and community integration projects globally, where sample sizes are small and impact limited [109, 130], as opposed to the larger treatment and risk assessment studies where the programmes and bodies of work are based on similar theoretical constructs [131]. This means, methodologically speaking, that large scale randomised controlled trials may not be the most effective method for understanding individual change and effective risk management [132], with the best projects potentially being a combination of quantitative and qualitative research to give a rounded view.

A final issue with the “success” criterion in integration is that it is not based on success, but on failure. We currently measure an individual’s success by whether they re-offend, not by how they have changed their lives or progressed along a desistance pathway [68]. In redeveloping KPIs we need to make sure that pro-social, progressive ones are included [36, 109], and understand the difference between a lack of reoffending and the development of a genuine commitment to desistance [133].

## Developing Fit for Purpose Policies and Practices

Criminal justice policies are usually reactionary in nature [46, 59] and can be poorly thought out in respect to cost or the challenges of implementation [3]. This is particularly true of sexual offense policies. In taking an EpiCrim approach, we need to reconsider the policy development and implementation on the community integration of people convicted of a sexual offence. All too often, policy development in this area is based on the impact of high-profile cases [for instance, Adam Walsh and Jacob Wetterling in the USA, Sarah Payne in the UK], public opinion, and media perspectives, with little consideration of the evidence base [134•, 135]. It would be safer to say that sexual offence policy tends to be evidence informed or ideologically driven rather than evidence based [15, 46, 136]; therefore, we need to take a more nuanced approach. In doing this, we need to be more client centred. In our field, when we are talking about patient or client involvement, we are discussing people who have either been a victim of or perpetrated sexual abuse [137••]; although, neither are central to the policy making process. Although the views of victim/survivors shape the development of sexual offense policy and practice [138], these tend to be those perspectives that fit with the pre-existing policy and practice positions. Alternative victim/survivor views—such as support for progressive measures including restorative measures [139, 140] or Circles of Support and Accountability [109]—are often marginalised. The development of informed, evidence-based, and nuanced community integration practice must come with an upskilling and education of the public in general, and policy makers specifically, about the complexity of sexual abuse. It is important to develop policy that enables desistance, rather than results in the individual failing, breaching, relapsing, and ultimately re-offending. Sexual abuse policy must enable good practice in rehabilitation and integration to occur, not stifle it. Therefore, we need a dual track policy and practice process that can be reactive and thoughtful. This means an ongoing process of research, development, and review. Community integration policy and practice needs to be constant and evolving, and not, as it is currently, sporadic and reactionary.

## Conclusion

The integration of people who have committed sexual offences back into the community is a difficult balancing act between risk management, public protection, and community relations. What we have seen in recent years, in line with the evolution of our understanding of general offending behaviour, is an emphasis on sexual offending as a health, life course, and well-being issue. This emphasises the importance of focusing on the person rather than the offence and, therefore, of partnership in the way that we collectively integrate people who have committed a sexual offence back into the community. The changing landscape in the community integration of people convicted of a sexual offence opens opportunities for new partnerships, improved funding streams, and a concrete, coherent multi-disciplinary approach. The reality of this new landscape of community integration is that people who commit sexual offences come from communities; therefore, communities have a role to play in their ongoing risk management and desistance.
